# Effect of ALDH2 on Sleep Disturbances in Patients with Parkinson’s Disease

**DOI:** 10.1038/s41598-019-55427-w

**Published:** 2019-12-12

**Authors:** Chia-Yen Lin, Rwei-Ling Yu, Ruey-Meei Wu, Chun-Hsiang Tan

**Affiliations:** 1 Department of General Medicine, Kaohsiung Medical University Hospital, Kaohsiung Medical University, Kaohsiung, Taiwan; 2 Institute of Behavioral Medicine, College of Medicine, National Cheng Kung University, Tainan, Taiwan; 3 Institute of Allied Health Sciences, College of Medicine, National Cheng Kung University, Tainan, Taiwan; 4 Department of Neurology, National Taiwan University Hospital, College of Medicine, National Taiwan University, Taipei, Taiwan; 5 Department of Neurology, Kaohsiung Medical University Hospital, Kaohsiung Medical University, Kaohsiung, Taiwan; 6 Graduate Institute of Clinical Medicine, College of Medicine, Kaohsiung Medical University, Kaohsiung, Taiwan

**Keywords:** Behavioural genetics, Neurodegeneration, Parkinson's disease

## Abstract

Monoamine neurotransmitters play essential roles in the regulation of arousal and sleep. Impaired metabolism of monoamine neurotransmitters could result in the accumulation of neurotoxic aldehyde metabolites and, hence, neuronal degeneration. Aldehyde dehydrogenases play an important role in the metabolism of the neurotoxic aldehyde metabolites, including the aldehyde metabolites of dopamine, serotonin, and noradrenaline. Deficient aldehyde dehydrogenase 2 (ALDH2) has been suggested to result in the accumulation of these biogenic aldehydes. An *ALDH2* single nucleotide polymorphism (SNP), rs671 (A), results in significantly reduced ALDH2 enzyme activity. A total of 83 Parkinson’s disease (PD) patients were recruited in this study. In addition to the genotypes of rs671, the patients were assessed with the PD sleep scale-2nd version (PDSS-2) and the Epworth sleepiness scale (ESS) for symptoms of daytime and nocturnal sleep disturbances. The patients carrying rs671 (A) had more frequent dozing while lying down to rest in the afternoon (ESS item5) (F = 7.308, p = 0.008) than the rs671 (GG) patients. The patients with rs671 (A) reported a trend toward more frequent difficulty staying asleep than the patients with rs671 (GG). (F = 3.278, p = 0.074). The results indicate that patients carrying allele rs671 (A) are more likely to experience impairment in the regulation of arousal and sleep. The results also support the hypothesis that the accumulation of neurotoxic monoamine neurotransmitter aldehyde metabolites secondary to reduced ALDH2 enzyme activity may cause more severe monoaminergic neuronal loss and, hence, more severe symptoms in the regulation of wakefulness and sleep.

## Introduction

Parkinson’s disease (PD) is a common neurodegenerative disorder. A meta-analysis of the worldwide data showed increasing prevalence with age, and the prevalence was suggested to be 1,087 and 1,903 per 100,000 persons among those between 70 to 79 years of age and those above 80 years of age, respectively^1^. Although Parkinson’s disease is traditionally characterized by the motor symptoms, non-motor symptoms were shown to cause more significant impairment in the quality of life (QoL), with symptoms of sleep and fatigue highly prevalent and strongly predictive of QoL change among patients with PD^2^. Around two-thirds of patients with PD report insomnia or are classified as poor sleepers, and around 30% experience excessive daytime sleepiness (EDS)^3,4^.

Although the pathological change in substantia nigra pars compacta (SNpc) is the hallmark of motor symptoms in PD, the pathological change responsible for the sleep-related disorders in patients with PD remains mostly unclear. Some studies suggested the pathological changes responsible for the sleep disorders may precede and are unrelated to the changes in substantia nigra^5^. Supporting evidence includes the research demonstrating that rapid eye movement sleep behavior disorder (RBD) may precede the motor symptoms of PD by decades^6^, suggesting that the pathological changes develop earlier than nigral dopaminergic neuronal degeneration. Furthermore, another study showed that the locus coeruleus-subcoeruleus complex, the arousal-modulating neuron groups, is affected earlier than the SNpc^7^. In recent years, it has been shown that monoaminergic neurotransmitters are involved in the regulation of the sleep-wake system, and monoaminergic neuronal degeneration causes sleep disorders^8^. Oxidative deamination of monoamine neurotransmitters results in the production of a number of neurotoxic aldehydes, which may accumulate in the central nervous system if not metabolized adequately by enzymes within the group of aldehyde dehydrogenases^9^. Among the aldehyde dehydrogenases, aldehyde dehydrogenase 2 (ALDH2) is of critical importance. It is well known that ALDH2 deficiency causes significant impairment in the metabolism of acetaldehyde, the accumulation of which causes alcohol-flush syndrome^10^. ALDH2 deficiency also causes impairment in the metabolism of biogenic aldehydes. The biogenic aldehyde products of dopamine, noradrenaline, and serotonin through the metabolism of monoamine oxidase (MAO) are 3,4-dihydroxyphenylacetaldehyde (DOPAL), 3,4-dihydroxyphenylglycolaldehyde (DOPEGAL) and 5-hydroxyindole-3-acetaldehyde (5-HIAL), respectively. These monoaminergic aldehydes may accumulate under the circumstances of ALDH2 deficiency^11,12^. An Asian-specific ALDH2 single nucleotide polymorphism (SNP), rs671 (A), causes an amino acid substitution from glutamic acid to lysine, and results in the greatly reduced enzyme activity of the protein product of *ALDH2* gene^13^. Almost half of the population in south Han Chinese and Japanese carries the impaired enzyme phenotype^14^.

Results obtained from both *in vitro* and *in vivo* experiments suggest that the accumulation of these monoamine-degraded aldehydes can lead to neurotoxicity^12,15–17^. Compatible with the findings suggestive of the neurotoxicity due to accumulation of monoamine neurotransmitter metabolites, our previous study showed that patients with PD carrying rs671 (A) allele showed a lower score in the mini-mental state examination (MMSE), higher frequency in cognitive impairments and deterioration in hobbies, and more severe disorganization and hypersexuality than patients without the (A) allele^18^.

In this study, we investigated whether ALDH2 deficiency secondary to rs671 (A) can cause sleep disturbances in patients with PD to gain a deeper understanding of the impact of monoamine neurotransmitters in the sleep-awake system regulation and the effect of rs671 (A) on the non-motor symptoms of PD patients.

## Results

A total of 41 patients with the genotype of rs671 (GG) and 42 patients with the genotype of rs671 (AG) or (AA) were included in the study. The allele frequency of rs671 (A) was 31.3% (52 out of 166). The genotype frequencies of the patients were in accordance with Hardy-Weinberg equilibrium (χ^2^ = 0.896, p = 0.344). There were no significant differences in age, gender, education years, age at onset^19,20^, disease duration, levodopa equivalent daily dose (LED), levodopa equivalent dose attributable to dopamine agonist, disease severity (Hoehn and Yahr stage, H&Y stage) and severity of motor symptoms (the Movement Disorder Society-Unified Parkinson’s Disease Rating Scale, MDS-UPDRS part I, II, III) between the two groups (Table 1).

**Table 1:** Demographic and Clinical Characteristics of the Study Groups.

	rs671 (GG) (n = 41)		rs671 (AG) + (AA) (n = 42)		Statistic	p Value
	mean	SD	Mean	SD		
Age (years)	64.937	8.5353	64.870	8.8799	t = 0.035	0.972
Gender (female/male)	15/26	—	20/22	—	χ^2^ = 1.036	0.309
Education (years)	12.146	3.9215	10.690	4.2512	U = 695.000	0.121
Age at Onset (years)	58.683	9.4113	58.500	9.3313	U = 848.000	0.906
Disease duration (months)	72.439	43.8532	76.286	38.0002	t = −0.427	0.670
Levodopa equivalent dose	731.427	417.3687	736.226	375.0122	t = −0.055	0.956
Levodopa equivalent dose attributable to dopamine agonist	99.573	98.1356	119.345	109.0187	U = 760.000	0.354
MMSE	28.244	1.5456	28.071	1.7305	U = 824.000	0.730
Hoehn and Yahr stage	2.073	0.6852	2.381	0.7949	U = 672.500	0.059
I	17.1%	—	11.9%	—	—	—
II	61.0%	—	45.2%	—		
III	19.5%	—	35.7%	—		
IV	2.4%	—	7.1%	—		
sum	100.0%	—	100.0%	—		
MDS-UPDRS part I (mentality)	7.805	5.4278	6.595	5.0609	t = 1.050	0.297
MDS-UPDRS part II (daily activities)	9.854	7.5682	9.381	6.7714	U = 854.000	0.949
MDS-UPDRS part III (motor)	25.854	12.1708	25.786	12.3022	U = 845.500	0.888
RBD with/without	20/21	—	16/26	—	χ^2^ = 0.964	0.326
**Age at onset, divided into three groups, proposed by Mehanna** ***et al***.^19^						
AAO ≦ 49	7	—	7	—	χ^2^ = 0.479	0.787
50 ≦ AAO ≦ 69	27	—	30	—		
AAO ≧ 70	7	—	5	—		
**Age at onset, divided into two groups, proposed by Wickremaratchi** ***et al***.^20^						
AAO < 49	6	—	5	—	χ^2^ = 0.002	0.966
AAO ≧ 49	35	—	37	—		

Abbreviations: SD, standard deviation; AAO, age at onset; MMSE, Mini-mental state examination, MDS-UPDRS, Movement Disorder Society-Unified Parkinson’s disease rating scale.

**Figure 1:**
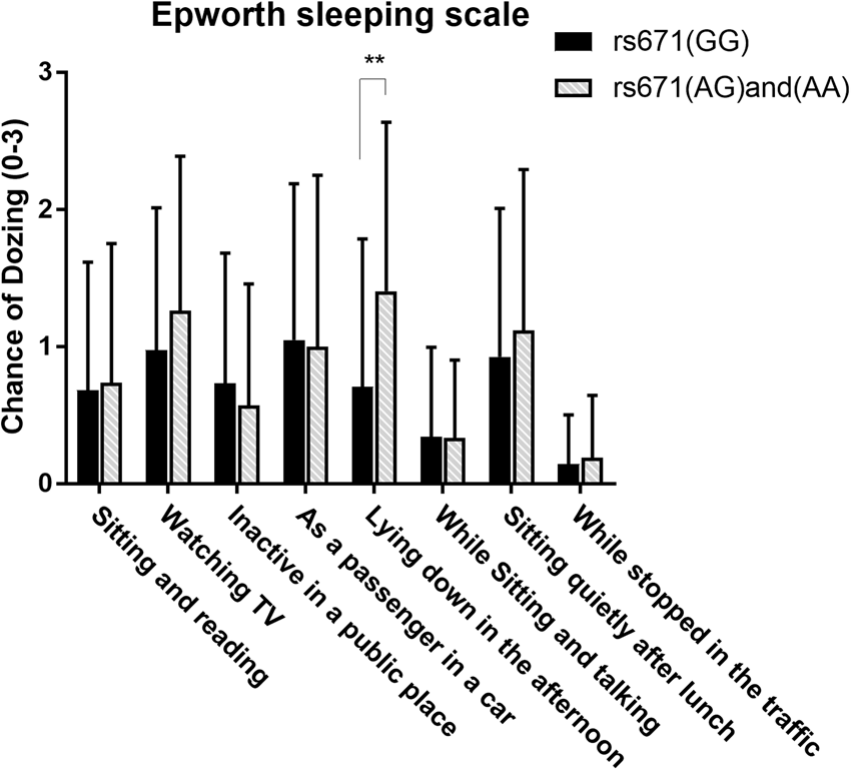
The means and standard deviations of each item on the Epworth sleeping scale in the 2 study groups. Patients with rs671 (AG) and (AA) showed significantly more frequent chance of dozing while “lying down in the afternoon when circumstances permit” (F = 7.308, p = 0.008). **Indicate p < 0.01.

In terms of daytime sleepiness, which was evaluated with Epworth sleepiness scale (ESS) scale, there was a significant difference in the ESS item 5 (Table 2). The patients carrying rs671 (A) had more frequent dozing while lying down to rest in the afternoon (ESS item5) (F = 7.308, p = 0.008) than the rs671 (GG) patients (Fig. 1). The percentage of patients with the sum of ESS ≧ 10 in the group carrying rs671 (A) is 28.6% (12 out of 42), and the percentage in the group carrying only rs671 (G) is 19.5% (8 out of 41). Although the percentage of patients with excessive daytime sleepiness (the sum of ESS ≧ 10) is higher in the group carrying rs671 (A), there was no statistical significance between the two study groups (χ^2^ = 0.502, p = 0.479).

**Table 2:** The Chinese version of the Epworth sleeping scale in the 2 study groups.

	rs671 (GG) (n = 41)		rs671 (AG) and (AA) (n = 42)		Mann-Whitney U	p-Value	F of Qaude’s test	P-value of Quade’s test
	mean	SD	mean	SD				
ESS1	0.683	0.9338	0.738	1.0136	U = 857.000	0.967	0.006	0.939
ESS2	0.976	1.0365	1.262	1.1275	U = 739.500	0.247	1.387	0.242
ESS3	0.732	0.9493	0.571	0.8874	U = 765.500	0.327	1.072	0.304
ESS4	1.049	1.1391	1.000	1.2494	U = 810.000	0.616	0.316	0.576
ESS5	0.707	1.0781	1.405	1.2309	U = 600.000	0.010	7.308	0.008**
ESS6	0.341	0.6561	0.333	0.5703	U = 848.000	0.880	0.020	0.889
ESS7	0.927	1.0814	1.119	1.1729	U = 795.500	0.526	0.391	0.534
ESS8	0.146	0.3578	0.190	0.4547	U = 840.500	0.767	0.109	0.742
ESS sum	5.561	4.9348	6.619	5.3827	U = 776.000	0.436	0.567	0.453
EDS (with/without)	8/33		12/30		χ^2^ = 0.502	0.479	—	—

**Figure 2:**
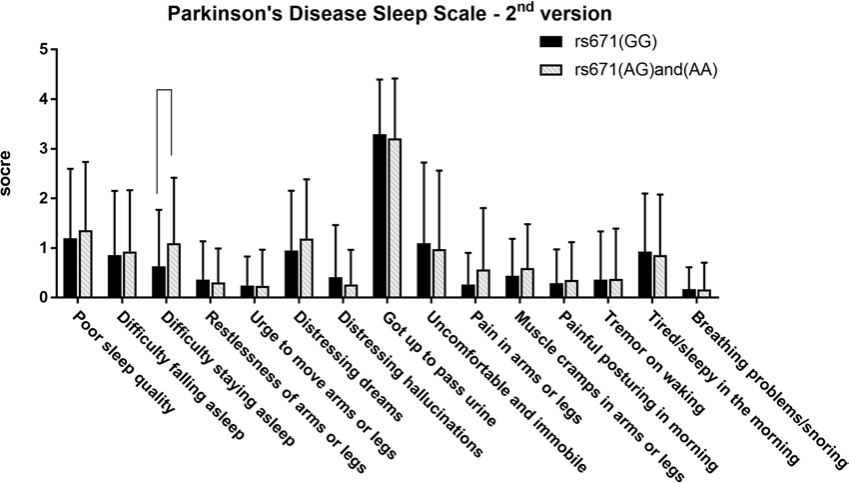
The means and standard deviations of each item on the Parkinson’s disease sleep scale-2^nd^ version in the 2 study groups. Patients with rs671 (AG) and (AA) showed a trend toward more frequent “difficulty staying asleep” (F = 3.278, p = 0.074).

With regard to nocturnal disturbances, there was a trend toward statistical significance in the PD sleep scale-2nd version (PDSS-2) item 3, “difficulty staying asleep” (Table 3). The results indicate that patients carrying rs671 (A) tend to more frequently report difficulty staying asleep than the patients with the genotype of rs671 (GG) (F = 3.278, p = 0.074) (Fig. 2). There was no significant difference in the proportion of patients diagnosed with RBD between the two groups with different genotypes (Table 1).

**Table 3:** Parkinson’s disease sleep scale-2^nd^ version in the 2 study groups.

	rs671 (GG) (n = 41)		rs671 (AG) and (AA) (n = 42)		Statistic	p- Value	F of Qaude’s test	P-value of Quade’s test
	mean	SD	mean	SD				
PDSS1	1.195	1.4003	1.357	1.3761	U = 806.500	0.599	0.301	0.585
PDSS2	0.854	1.2954	0.929	1.2375	U = 805.500	0.578	0.262	0.610
PDSS3	0.634	1.1348	1.095	1.3217	U = 686.000	0.073	3.278	0.074
PDSS4	0.366	0.7667	0.310	0.6803	U = 811.000	0.535	0.427	0.515
PDSS5	0.244	0.5823	0.238	0.7262	U = 836.500	0.724	0.082	0.775
PDSS6	0.951	1.2031	1.190	1.1943	U = 752.500	0.293	1.063	0.306
PDSS7	0.415	1.0482	0.262	0.7005	U = 831.500	0.671	0.245	0.622
PDSS8	3.293	1.1010	3.214	1.2003	U = 832.000	0.758	0.151	0.698
PDSS9	1.098	1.6249	0.976	1.5848	U = 805.000	0.554	0.522	0.472
PDSS10	0.268	0.6334	0.571	1.2325	U = 807.000	0.494	0.458	0.500
PDSS11	0.439	0.7433	0.595	0.8851	U = 772.500	0.346	0.845	0.361
PDSS12	0.293	0.6798	0.357	0.7594	U = 839.000	0.776	0.079	0.779
PDSS13	0.366	0.9684	0.381	1.0110	U = 858.500	0.972	0.000	0.988
PDSS14	0.927	1.1703	0.857	1.2212	U = 810.500	0.615	0.198	0.658
PDSS15	0.171	0.4417	0.167	0.5372	U = 824.000	0.551	0.305	0.583

## Discussion

To the best of our knowledge, this is the first study investigating the effect of ALDH2 on sleep disturbances in patients with PD, including EDS and nocturnal disturbances. In general, our results indicate that the patients carrying rs671 (A) allele are more likely to develop excessive daytime sleepiness and also tend to have difficulty maintaining asleep than those carrying only rs671 (G) allele.

Previous studies reported the prevalence of EDS among patients with PD to be 16–74%, and usually around 33%^3^. In this study, the EDS rate was higher in patients carrying rs671 (A) (28.6%) than patients with rs671 (GG) (19.5%), although there was no significant difference (χ^2^ = 0.502, p = 0.479). The EDS was proposed to be associated with LED, and dopamine agonist was suggested to have an impact on sleep attack^21^. The LED, as well as the LED attributable to dopamine agonists, were comparable between the two groups in this study. As described in our previous study, evaluating daytime sleepiness with ESS might underestimate the prevalence of sleep disturbances in Taiwan^4^, so it is possible that the prevalence of EDS may also be underestimated in this study.

In the PDSS-2 questionnaire, patients carrying rs671 (A) allele tend to more frequently report difficulty maintaining asleep. Nonetheless, despite the potential difference in maintaining asleep at night, other items on PDSS-2 failed to reveal a significant difference between the two groups. The result suggests that ALDH2 deficiency contributes specifically to the difficulty in sleep maintenance, which can be due to a perturbed circadian rhythm or an unstable sleep-awake transition. In addition, the item “get up to pass urine” had the highest score in both groups, and the finding is compatible with previous results indicating frequent need to pass urine at night as one of the leading causes of sleep disturbances among patients with PD^3^.

In the present study, we utilized Quade’s test for controlling the impact attributable to confounding variables (age, age at onset, and LED). The analysis made the result of ESS item 5 more significant after controlling the confounders.

The A allele of *ALDH2* rs671 SNP causes an amino acid substitution from glutamic acid to lysine, resulting in the significantly reduced enzyme activity. ALDH2 is a tetrameric enzyme, and one study reported that one inactive subunit encoded by the SNP rs671 (A) results in the inactivation of the tetramer enzyme. Hence, the percentage of ALDH2 enzyme with four active subunits in heterozygous individuals through the random association of the active and inactive subunits would be 6%^22^. One more recent study showed that the tissue ALDH2 activity in individuals with rs671(AG) was 17% that of individuals with rs671 (GG), while the activity in individuals with rs671(AA) was too low to be determined precisely^23^. The reduced enzyme activity results in the accumulation of aldehydes, and is responsible for the well-known Asian flush and can even cause alcohol-related cancers as a result of accumulation of acetaldehyde^12,24,25^.

ALDH2 also plays a major role in the metabolism of monoamine neurotransmitters. The oxidative deamination of dopamine, norepinephrine, and serotonin by MAO results in the production of DOPAL, DOPEGAL, and 5-HIAL, respectively. These aldehydes have been shown to be neurotoxic with both *in vitro* and *in vivo* experiments, and the crucial role of ALDH2 in the metabolism of these aldehydes into non-toxic metabolites has been proposed^26–28^. At the concentration close to the physiological levels reported in healthy human postmortem specimens of the substantia nigra^29^, DOPAL caused reduced viability of PC12 cells, a cell line with dopaminergic properties, by around 30%^30^. DOPAL has also been shown to be toxic to cell lines of neuron-like cells, SH-SY5Y^31^ and SK-N-SH cells^32^, and neurons from fetal rat mesencephalic tissues and neostriatal synaptosomal preparations^33^. Stereotaxic injection of DOPAL in the substantia nigra caused selective loss of dopaminergic neurons in the substantia and triggered a behavioral phenotype consistent with other PD animal models^34^. The neurotoxicity of DOPEGAL has also been demonstrated across different studies, in which DOPEGAL was shown to cause a concentration- and time-dependent toxicity to PC-12 cells at concentrations as low as 5.9 μM^35^. Injection of DOPEGAL to rostral ventrolateral medulla (RVLM) in amounts as low as 50 ng was shown to cause dose- and time-dependent loss of adrenergic neurons^36^. Furthermore, the aldehyde metabolite of serotonin, 5-hydroxyindole-3-acetaldehyde (5-HIAL), accumulates in the presence of daidzin, an ALDH2 inhibitor^37^, and has been shown to cause oligomerization of alpha-synuclein like DOPAL^17^.

Although studies suggest that glutamate and gamma-Aminobutyric acid (GABA) have more direct and immediate impact on the regulation of wakefulness and sleep, the monoaminergic neurotransmitters have been shown in numerous studies to play essential roles in the arousal promoting system and sleep-promoting system^8,38^. Noradrenergic neurons in the locus coeruleus (LC) have been shown to discharge tonically at highest frequency during wakefulness and to reduce their activity during non-rapid eye movement (NREM) sleep with virtually absent activity during rapid eye movement (REM) sleep^39^. By applying optogenetic tools in mice, photoinhibition of locus coeruleus neurons was shown to cause a reduction in the duration of wakefulness, while photostimulation of locus coeruleus neurons caused immediate sleep-to-wake transitions^40^. Besides, photoinhibition of LC neurons during stimulation of Hypocretin (Hcrt) neurons was shown to block Hcrt-mediated sleep-to-wake transitions, while photostimulation of LC neurons with concomitant photostimulation of Hcrt neurons significantly increased the probability of sleep-to-wake transitions compared with stimulation of Hcrt neurons alone^41^. All the results above indicate the crucial role of noradrenergic neurons in LC as an effector in the regulation of wakefulness.

In addition to noradrenergic neurons, dopaminergic neurons in the ventral tegmental area (VTA) and dorsal raphe nucleus (DRN) have also been found to be involved in the regulation of wakefulness or sleep. VTA dopaminergic neurons were found to be activated during REM sleep, and this activation was similar to the activity measured during the consumption of palatable food^42^. With fiber photometry to record calcium activity, the activity of VTA dopaminergic neurons was found to be reduced during NREM sleep compared with both wakefulness and REM sleep^43^. Optogenetic activation of VTA dopaminergic neurons was found to initiate and maintain wakefulness, while chemogenetic inhibition of VTA dopaminergic neurons decreased wakefulness and promoted sleep^43^. Optogenetic stimulation and chemogenetic inhibition of DRN dopaminergic neurons were shown to promote and reduce wakefulness, respectively^44^. Furthermore, the importance of dopamine is also evidenced by the results showing that optogenetic activation of nucleus accumbens dopamine D_1_ receptor (D_1_R)-expressing neurons induced immediate transitions from NREM sleep to wakefulness, and chemogenetic stimulation prolonged arousal^45^. These results all indicate the importance of dopamine in the regulation of wakefulness. Moreover, serotonergic DRN neurons were shown to be active during wakefulness and progressively less active during the early, middle and late phases of slow-wave sleep^46^. Meanwhile, the involvement of serotonin in the regulation of sleep is also evidenced by the finding showing increased REM sleep and decreased serotonin level with DRN microdialysis perfusion of 8-Hydroxy-2-(Di-n-Propylamino)Tetralin (8-OH-DPAT), a selective hydroxytryptamine (5-HT) receptor agonist that inhibits serotonergic neural activity via autoreceptor-mediated inhibition^47^. At the same time, the sleep-promoting ventral lateral pre-optic nucleus (VLPO) innervates the monoaminergic arousal components (including LC and raphe system) and receives inputs from these areas, reciprocally. Dysfunction of the monoaminergic neurons may hence cause disturbance in the sleep-promoting system as well^8,48^.

Degeneration of monoaminergic neurons is the histopathological hallmark of PD. Loss of noradrenergic neurons in LC and dopaminergic neurons in VTA has been reported across several studies in patients with PD^49–53^. Neuronal degeneration in DRN has also been reported in patients with PD, although whether dopaminergic or serotonergic neurons are more severely affected remain unknown^52^. The monoaminergic neuron degeneration may be the underlying cause responsible for the symptoms of excessive daytime sleepiness and sleep disturbances in patients with PD. Moreover, in the present study, the results demonstrate the differences in the profiles of sleep disorders between the two groups with different rs671 genotypes. The findings support the hypothesis that the accumulation of neurotoxic monoamine neurotransmitter aldehyde metabolites as a result of inadequate ALDH2 enzyme activity can cause more severe monoaminergic neuronal loss and more severe disorders in the regulation of wakefulness and sleep.

The present study has potential limitations. The participants in the study were recruited from tertiary medical centers and may cause selection bias. In addition, to ensure accurate and correct report of the sleep symptoms, PD patients who scored < 24 on MMSE were excluded from the study. On the other hand, patients with more severe sleep disturbances may not be included. Another drawback of this study is the need for combining the results of patients with rs671 (AG) and rs671 (AA) into one group for analysis because the genotype frequency of rs671 (AA) is low and hence the number of patients carrying rs671 (AA) in this study is small. The number of patients carrying rs671 (AA) is much smaller (n = 10) than that of the rs671 (GG) (n = 41) and rs671 (AG) (n = 32). If the three groups are analyzed separately, the possibility of type I error would be too high. The study also did not quantify for doses of sleep-aids. This study also did not apply polysomnography (PSG) to obtain objective parameters for sleep evaluation. Utilizing PSG may provide more information and help differentiate REM sleep from NREM sleep because poor sleep maintenance reported by the patients might reflect an increase in REM sleep bout.

To conclude, the present study is the first study investigating the association between sleep disturbances in patients with PD and *ALDH2* SNP rs671. The results indicate that PD patients with allele rs671 (A) are more likely to have excessive daytime sleepiness and may tend to have difficulty maintaining asleep, and provide evidence suggesting that ALDH2 may modulate the accumulation of monoamine neurotransmitters and hence, the non-motor symptoms of patients with PD.

## Methods

### Participants

This study included 83 patients who were diagnosed with idiopathic PD according to the United Kingdom PD Society Brain Bank clinical diagnostic criteria^54^. These patients were referred from neurologists from the outpatient departments from three medical centers located in three different cities in Taiwan. We included patients with the following features: mentally able to complete the interview, self-report questionnaires, and neuropsychological tests (scored ≧ 24 in the mini-mental state examination). The neuropsychological tests were completed by the patients themselves and with some assistance from the investigators if needed. We excluded patients with the following features: atypical parkinsonism, illiteracy, history of brain surgery, or any severe systemic disease.

We obtained all of the written informed consent from the participants before enrollment, following the ethical standards outlined in the 1964 Declaration of Helsinki. All study procedures were approved by the ethical research committee of Kaohsiung Medical University Hospital, National Cheng Kung University Hospital, and National Taiwan University Hospital and all methods were performed in accordance with the approved guidelines. The detailed information of demographic, dopamine replacement therapy and motor severity was obtained from the medical record assessed by the neurologists. Levodopa equivalent dose, as well as Levodopa equivalent dose attributable to dopamine agonist of each participant, was calculated according to the method of Tomlinson *et al*.^55^. Motor symptoms and severity were assessed according to Movement Disorder Society-Unified Parkinson’s Disease Rating Scale (MDS-UPDRS)^56^ and Hoehn and Yahr staging criteria^57^, respectively.

### Assessment of symptoms

We assessed the 83 PD patients with MMSE, MDS-UPDRS, H&Y staging to investigate the subgroup characteristics of the mental, motor and daily activities. We obtained the history of RBD (Rapid eye movement sleep behavior disorder) from the patient’s sleep companion. We used two sleep-related questionnaires, including the Chinese version of the Epworth sleeping scale (ESS), the Parkinson’s Disease Sleep Scale -2nd version (PDSS-2)-Taiwan form.

ESS is a useful tool to evaluate daytime sleepiness. Each item scored from 0 to 3, with 0 beings “would never doze” and 3 being “ high chance of dozing”. If a total score of the eight items yields ten or more, the patient is categorized as suffering from excessive daytime sleepiness (75% sensitivity and 82.4% specificity for daytime sleep episodes)^58^. The Chinese version of the ESS is reliable (Cronbach’s α = 0.81, test-retest reliability = 0.74)^59^.

The English PDSS-2 consists of 15 questions about various sleep and nocturnal disturbances which are to be rated by the patients using one of five categories, from 0 beings “very often” to 4 being “never” in question 1; from 0 being “never” to 4 being “very often” in question 2 to 14. The total score ranges from 0 (no disturbance) to 60 (maximum nocturnal disturbance). The Chinese version had a totally opposite scoring, from 0 beings “never” to 4 beings “very often” in question 1, from 0 beings” very often” to 4 being “never” in question 2 to 14. We replaced the original score (obtained from the patient’s score in Chinese questionnaire) to a final score of “4 minus original score”. PDSS-2 is a test of good reliability (Cronbach’s α = 0.73, test-retest reliability intra-class-coefficient = 0.80)^60^.

### Genetic analysis

We performed blood sampling of the 83 patients’ genomic DNA. We extracted genomic DNA from peripheral blood leukocytes with the Genomic DNA Extraction Kit (Geneaid, New Taipei City, Taiwan). ALDH2 rs671 SNP genotype was determined with TaqMan probes and the StepOnePlusTM system with the StepOne software (Applied Biosystems, Grand Island, NY, USA). The laboratory technicians, who were blinded to the patient’s demographic characteristics, performed the genotyping, and read the results.

### Statistical analysis

We examined each item mentioned earlier based on the 83 patient’s ALDH2 rs671 genotype. Proportions were calculated for qualitative variables, and means and standard deviations (SDs) were calculated for quantitative variables. Because there were only ten of the participants carrying the rs671 (AA) genotype, we combined Patients with genotype rs671 (AG) and rs671 (AA) into one group. This combination is valid, based on the study reporting the rs671 (AA)-encoded enzymes is completely inactive and the rs671 (AG)-encoded enzyme is partially inactive (16~17% of activity of that of the rs671 (GG))^23^. We applied the Kolmogorov-Smirnov test for testing normality. Afterward, we tested quantitative variables with the t-test or Mann-Whitney U test and tested qualitative variables by using chi-squared test. We controlled the impact attributable to confounding variables (age, age at onset and LED) by applying Quade’s test. Statistical significance was predetermined with an alpha level less than 0.05. The statistical analysis was done by a commercially available software program (IBM Corp. Released 2012. IBM SPSS Statistics for Windows, Version 21.0. Armonk, NY: IBM Corp.).
